# Recruitment of the Histone Variant MacroH2A1 to the Pericentric Region Occurs upon Chromatin Relaxation and Is Responsible for Major Satellite Transcriptional Regulation

**DOI:** 10.3390/cells12172175

**Published:** 2023-08-30

**Authors:** Ludmila Recoules, Nicolas Tanguy Le Gac, Fatima Moutahir, Kerstin Bystricky, Anne-Claire Lavigne

**Affiliations:** 1 Centre de Biologie Intégrative (CBI), MCD, Université de Toulouse Paul Sabatier, UPS, Université de Toulouse, UT, CNRS, F-31062 Toulouse, France; ludmila.recoules@inserm.fr (L.R.); nicolas.tanguy-legac@univ-tlse3.fr (N.T.L.G.); fatima.moutahir@univ-tlse3.fr (F.M.); 2 Institut Universitaire de France (IUF), F-75231 Paris, France

**Keywords:** pericentromeric regions, histone variant, macroH2A1, chromatin decondensation

## Abstract

Heterochromatin formation plays a pivotal role in regulating chromatin organization and influences nuclear architecture and genome stability and expression. Amongst the locations where heterochromatin is found, the pericentric regions have the capability to attract the histone variant macroH2A1. However, the factors and mechanisms behind macroH2A1 incorporation into these regions have not been explored. In this study, we probe different conditions that lead to the recruitment of macroH2A1 to pericentromeric regions and elucidate its underlying functions. Through experiments conducted on murine fibroblastic cells, we determine that partial chromatin relaxation resulting from DNA damage, senescence, or histone hyper-acetylation is necessary for the recruitment of macroH2A1 to pericentric regions. Furthermore, macroH2A1 is required for upregulation of noncoding pericentric RNA expression but not for pericentric chromatin organization. Our findings shed light on the functional rather than structural significance of macroH2A1 incorporation into pericentric chromatin.

## 1. Introduction

Histone post-translational modifications, histone variants, DNA-binding factors, and architectural proteins regulate the three-dimensional (3D) chromatin organization and DNA-related processes [1,2]. Histone variants replace canonical histones in a locus-specific manner, which endows chromatin with properties required for nuclear functions [3]. The histone variant macroH2A1 (mH2A1) is a vertebrate-specific [4,5] histone H2A variant, composed of an N-terminal “H2A-like” domain (64% identical to H2A) and a “linker” domain that positions a C-terminal 25 kDa “macro” domain outside the nucleosome [6]. Expression of the highly conserved H2AFY gene produces two splicing isoforms, mH2A1.1 and mH2A1.2, that differ in a 30-amino-acid region within the macro domain [6]. mH2A1 is enriched at heterochromatin domains on the inactive X chromosome (Xi) [7,8] or on autosomes. Concerning the latter, mH2A1 forms large domains at facultative heterochromatin [9,10,11], to a lesser extent, at constitutive heterochromatin marked with the histone mark H3K9me3 [12], and at silent ribosomal DNA segments (rDNA) [13]. Moreover, in senescent cells, this histone variant is incorporated into Senescence-Associated Heterochromatin Foci (SAHFs) [14], which are composed of both heterochromatin types and pericentromeric DNA regions [15,16]. In quiescent human or mouse lymphocytes or cells treated with HDAC and DNMT1 inhibitors, mH2A1 was found to be recruited to pericentric regions [17,18]. While mH2A1 is associated with condensation of the Xi chromosome, mH2A1 is not essential for initiating or maintaining inactivation of this chromosome [7,19,20,21,22]. However, some genes present on the Xi chromosome were reactivated upon experimentally induced loss of mH2A1 when cells were treated with trichostatin A (TSA) [23]. At autosomes, loss of mH2A1 modified heterochromatin marks and gene expression only marginally [12,19,24,25,26]. The functions of mH2A1 at pericentric heterochromatin or within SAHFs have not yet been uncovered. In this study, we investigated the mechanisms underlying the recruitment of mH2A1 to pericentric regions and explored its functional significance in these genomic areas. Our findings reveal that senescence and various insults, such as induction of double-strand breaks (DSBs) and hyperacetylation, lead to a robust recruitment of mH2A1 to pericentric regions in murine fibroblast cells. Pericentric chromatin relaxation was a prerequisite for mH2A1 incorporation. Although mH2A1 was not required for this relaxation to occur, it is necessary for the activation of transcription of pericentromeric noncoding RNA, which has been suggested to be essential for heterochromatinization of pericentromeric regions [27].

## 2. Materials and Methods

**Cell Culture**. The L929 cell line was kindly provided by Jerome Cavaillé from CBI-Toulouse, France. MCF7 cell lines were purchased from ATCC and were maintained and amplified in Dulbecco’s Modified Eagle Medium (DMEM) for L929 and in DMEM-F12 for MCF-7 cells, supplemented with gentamycin (50 μg/mL) (Gibco, Waltham, MA, USA), fetal bovine serum (10%, Gibco), and sodium pyruvate (100 mM, Sigma-Aldrich, Burlington, MA, USA).

Cells were maintained in a humidified incubator at 37 °C with 5% CO2. Cell lines were regularly tested for mycoplasma infection (MycoAlert, Lonza, Basel, Switzerland). Senescence induction was carried out using 12.5 μM etoposide (Cell Signaling, Danvers, MA, USA #2200) over 24 h followed by 3–4 days of release [28,29]. Trichostatin A (TSA) treatment was carried out using 500 nM TSA (T8552, Sigma) over 48 h. ATM inhibitor treatment was carried out using 20 μM of Ku55933 inhibitor (Tocris Bioscience, Bristol, UK), added 1 h before Cas9-MajS transfection. The generation of L929 mH2A1 KO clones was carried out according to the procedure presented in [30]. gRNA and plasmids used are given in Table A1 and Table A2. After transfection of the two plasmids, Cas9_gRNA1 and Cas9-GFP_gRNA2, we carried out clonal selection with Puromycin followed by limiting dilution to obtain monoclonal clones.The expression of mH2A1 in selected clones was tested by Western blot, IF, and PCR-based screening strategy. We also selected these two clones using PCR-based screening strategy showing the presence of two different homozygous DNA mutations.

**Transfection and siRNA Knockdown**. At 30–50% confluence, transfection of siRNA (11 nM) against HIRA was performed using INTERFERin (Polyplus-Ozyme, Illkirch, France) according to the manufacturer’s protocol. Transfections of plasmids were carried out with FuGene HD (Promega, Madison, WI, USA) according to the manufacturer’s protocol. siRNA and plasmid sequences are available in Table A2 and Table A3, respectively. Cells were recovered two and three days post-plasmid and siRNA transfections, respectively. Knockdown efficiency was analyzed by Western blot.

**Real-Time qPCR**. Total RNA was isolated using the RNAeasy midi kit (Qiagen, Hilden, Germany) followed by digestion of residual genomic DNA by Invitrogen Turbo DNA-free kit. Purified RNA was reversed transcribed to cDNA using Maxima H Minus first Strand cDNA synthesis kit (Promega). The sequences of the primers used are available in Table A4. RT-PCR was performed using iTAq Universal SYBR Green (Bio-Rad, Hercules, CA, USA) according to manufacturer’s instructions. The relative expression levels of MajS ncRNA were normalized to GAPDH RNA expression and evaluated according to the 2${}^{-\Delta \Delta \mathrm{Ct}}$ method. The same method was used for senescence markers but normalized with 18S rRNA expression (Figure S3B).

**Western Blot Analysis**. Cells were lysed and subjected to Immunoblot analysis as previously described [31]. Briefly, protein extracts were separated in 10% polyacrylamide (1:125 bisacrylamide:acrylamide) SDS gels, transferred onto nitrocellulose membrane (Bio-Rad), and blocked with PBS-Tween 0.4%–Milk 5% for 1 h at room temperature (RT) with rotation. Membranes were then incubated with primary antibodies overnight (O/N) at 4 °C for 1 h 30 m at RT in PBS-Tween 0.4%–Milk 5% with rotation. Primary antibodies are described in Table A5. Membranes were next incubated with secondary antibody in PBS-Tween 0.4%–Milk 5% for 1 h at RT with rotation and the signal was detected using chemiluminescence. Secondary antibodies are described in Table A5. Signal quantifications were carried out with Image Lab software (v6.0) (Bio-Rad).

**Senescence-associated β-galactosidase assay**. Cells were treated with etoposide (Cell Signaling #2200) (12.5 μM) over 24 h followed by an etoposide release for 3–4 days. Senescence-associated β-galactosidase assay was then performed with Senescence β-Galactosidase Staining Kit (Cell Signaling #9860) according to manufacturer’s instructions. Cells were photographed using a light microscope at ×20 magnification (Invitrogen EVOS Digital Color Fluorescence Microscope, Carlsbad, CA, USA). Cell counting was carried out with ImageJ in ten different fields per condition. Two independent experiments were performed for each condition.

**Immunofluorescence and confocal Microscopy**. Two or three days post-transfection, cells were fixed with 4% paraformaldehyde for 10 min at RT. Cell permeabilization was carried out using 0.1% Triton X-100 in PBS for 10 min at RT. Cells were then blocked with 5% BSA-0.15% Tween in PBS for 1 h at RT. Next, cells were incubated with primary antibody O/N at 4 °C. Cells were then incubated with Alexa conjugated secondary antibody for 1 h at RT. Antibody references and dilutions are provided in S5 Table. The coverslips were finally incubated with Hoechst (Invitrogen, 33342) for 30 min and then mounted with mounting media (Vectashield). Images were acquired with Zeiss LSM 710 big confocal microscope using ×63 PL APO oil DIC On 1.4 objective for all experiments. Images were taken in Z-stacks with a voxel size of 300 nm. Max-intensity projection of Z-stacks are shown.

**Analysis of microscopy images**. Image analyses of chromocenters, other foci, and whole nuclei were performed using ImageJ (v.1.53t). Max-intensity projection images were used. Cells were selected based on Hoechst staining and chromocenters were defined as the Hoechst-dense regions. For the experiments using Cas9-MajS gRNA, positive cells were selected on the basis of the presence of Cas9-GFP and/or γH2AX foci at chromocenters. Different parameters were evaluated: area, mean intensity, perimeter, and circularity. For Figure S8E, whole-nucleus intensities of H3K9me3 and HP1α were measured and used to generate boxplots. For Figure S4C, whole-nucleus intensities of mH2A1 were measured when mH2A1 did not form foci at chromocenters. Per cell, we calculated the mean of the different parameters (area, mean intensity, perimeter, and circularity) measured for each focus. Boxplots were generated using R studio. The number of cells presenting foci of mH2A1-, γH2AX-, and SA-βgal-positive cells was counted manually from different biological replicates. Results are presented as mean ± SD. The number of cells presenting decondensed chromocenters was counted by eye, based on the comparison with chromocenter organization in untreated cells. The scatter plot was generated using R studio.

**Statistics and reproducibility**. Results from at least 2 biological replicates are presented except when stated otherwise in the figure legends.

Statistical analyses were performed using R or GraphPad Prism. Wilcoxon tests or t-test were used to assess the significance of the observed differences between samples. The figure legend provides information about the type of test that was employed. Differences were considered significant at a *p* value of 0.05 or less. **** *p*-value < 0.0001, *** *p* < 0.001, ** *p* < 0.01, * *p* < 0.05, ns: non-significant.

## 3. Results

### 3.1. The Histone Variant mH2A1 Accumulates at Pericentric Heterochromatin in Mouse Senescent Cells

**Figure 1:**
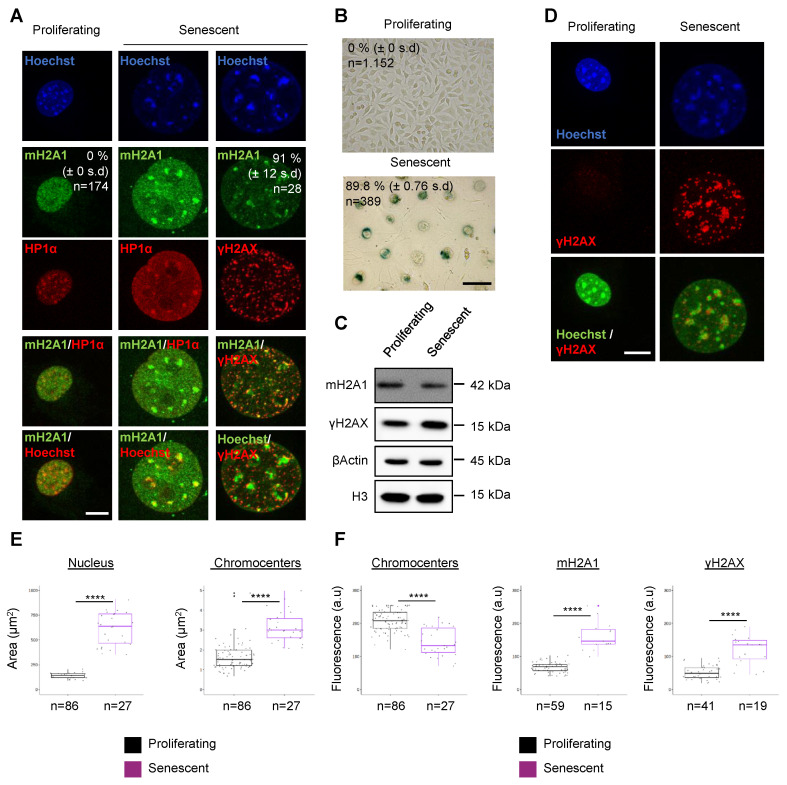
The histone variant mH2A1 is recruited to pericentric heterochromatin in mouse senescent cells. (**A**) Immunofluorescence (IF) confocal images of proliferating and senescent L929 mouse cells stained with Hoechst and antibodies specific for mH2A1, HP1α and γH2AX. Senescence was induced using etoposide treatment (12.5 μM, 24 h + 4 days of release). Percentage of cells presenting mH2A1 foci at pericentric regions is shown, represented as means ± standard deviation (SD) from two biological replicates. Scale bar = 10 μm. (**B**) Representative images of a senescence-associated β-galactosidase activity (SA-βgal) assessed by X-gal staining of proliferating and senescent cells. Percentage of SA-βgal-positive cells is indicated as means ± SD from two biological replicates. Scale bar = 100 μm. (**C**) Immunoblot analysis of mH2A1, γH2AX, β-actin, and H3 in total extract of proliferating and senescent cells. Apparent molecular weights are indicated. (**D**) IF confocal analysis of proliferating and senescent cells stained with Hoechst and antibody specific for γH2AX. Scale bar = 10 μm. (**E**) Quantifications of nuclear and chromocenter areas (Hoechst-dense labelling) in proliferating and senescent cells. (**F**) Quantifications of chromocenters (Hoechst-dense labelling), mH2A1, and γH2AX mean fluorescence intensities in proliferating and senescent cells. The number of cells analyzed for each condition is given (n). On boxplots, each point corresponds to the mean number of foci per cell, except for ‘nucleus’, where they represent the actual values. For statistical analysis, Wilcoxon tests were used to assess the significance of the observed differences. Differences were considered significant at a *p*-value of 0.05 or less. **** *p*-value < 0.0001.

During senescence induction in human cells, mH2A1 is known to be recruited to SAHFs that contain pericentric regions [14]. We asked if mH2A1 was also recruited to murine pericentric regions during senescence induction. Murine “chromocenters” offer the possibility to easily visualize the clustering of pericentromeric regions as large dense foci with Hoechst [32,33]. We therefore examined the localization of mH2A1 in proliferating murine L929 fibroblasts. mH2A1 presented a general faint staining throughout the nucleus but was not detectable at chromocenters (Figure 1A). We then treated L929 fibroblasts with 12.5 μM of etoposide, a topoisomerase II inhibitor, followed by an etoposide release over 3–4 days to induce senescent cells [28,29] (Materials and Methods). In addition to general diffuse nuclear staining, the most striking feature of mH2A1 distribution in senescent cells was a pronounced labeling of chromocenters, which co-localized with HP1α (Figure 1A). The percentage of cells showing pronounced pericentric mH2A1 staining (∼91% ± 12 SD) (Figure 1A) was in the same range as senescence-associated βgalactosidase-positive cells (SA-βgal) (89.8% (±0.76 SD)) (Figure 1B). Immunoblot analysis of mH2A1 protein levels indicated no increase in total mH2A1 protein in senescent cells compared to proliferative cells (Figure 1C), suggesting that the endogenous mH2A1 content was redistributed to pericentric regions upon senescence induction. Increased levels of phosphorylated histone H2AX (γH2AX) are consistent with the fact that senescent cells are known to permanently maintain a DNA damage response [34,35]. Therefore, we compared γH2AX staining intensities in proliferative and senescent mouse cells. γH2AX signals in proliferative cells were weaker than in senescent cells (Figure 1D). In senescent cells, in addition to small foci uniformly distributed throughout the nucleus, γH2AX formed larger foci, which partly co-localized with chromocenters. These larger foci also partly co-localized with mH2A1 at pericentric centers (Figure 1A). We noted that γH2AX foci were frequently found near the periphery of chromocenters, in agreement with the idea that DSBs within pericentric DNA relocalize to the periphery of chromocenters upon repair [36,37,38]. To assess the nature and extent of chromatin alterations in response to senescence induction, we measured the area and the average intensity of Hoechst, mH2A1, and γH2AX labeling (Figure 1E,F). We found that the nuclear volume increased drastically upon senescence induction (a ∼3-fold increase) (Figure 1E). Moreover, we discovered that chromocenter areas were larger in senescent cells compared to the ones in proliferative cells. Fluorescence intensity was decreased within the enlarged chromocenters (Figure 1F). These results indicate that chromocenters of mouse senescent cells are partially decondensed compared to chromocenters of proliferative cells. Surprisingly, chromatin relaxation was not accompanied by the eviction of HP1α (Figure 1A).

### 3.2. Cas9-Mediated Induction of DSBs at Pericentric Heterochromatin Triggers Recruitment of mH2A1

**Figure 2:**
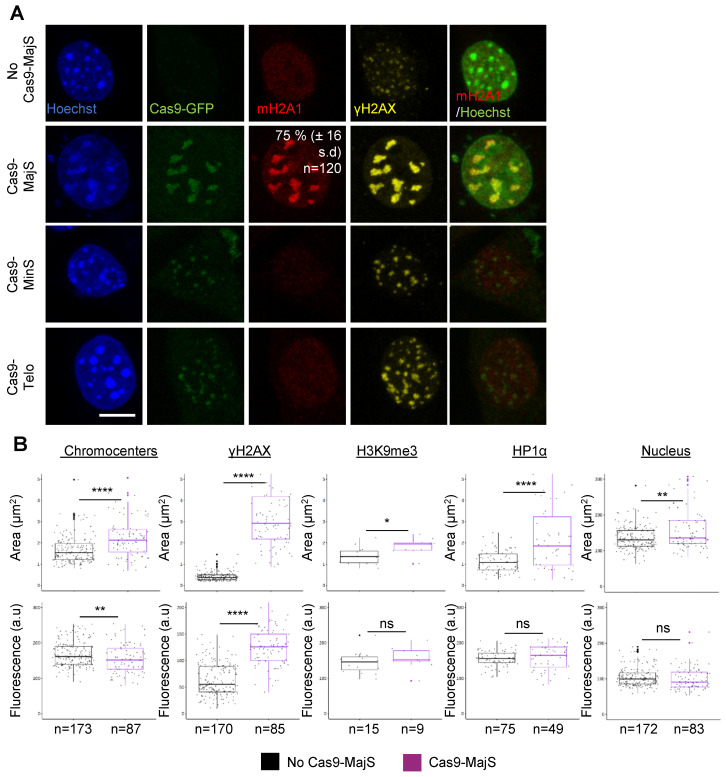
mH2A1 is recruited to Cas9-induced DSBs at pericentric regions. (**A**) IF confocal images of 48 h post-transfected cells co-expressing Cas9-GFP and gRNAs, stained with Hoechst and antibodies specific for mH2A1 and γH2AX. Three different gRNA are used: a gRNA targeting major satellites (MajS), corresponding to the pericentric DNA, a gRNA targeting minor satellites (MinS), corresponding to the centromeric DNA, and a gRNA targeting telomeres (Telo). Percentage of cells exhibiting mH2A1 foci at MajS are shown, represented as means ± SD from 6 biological replicates. Scale bar = 10 μm. (**B**) Quantifications of the mean areas and fluorescence intensities of chromocenters (Hoechst-dense labelling), γH2AX, H3K9me3, HP1α and nucleus (Hoechst labelling) in 48 h post-transfected negative (no Cas9-MajS) and positive cells (Cas9-MajS), taken from > 3 biological replicates, except for HP1α (2 biological replicates) and H3K9me3 (1 biological replicate). The number of cells analyzed for each condition is given (n). On boxplots, each point corresponds to the mean number of foci per cell, except for ‘nucleus’, where they represent the actual values. Wilcoxon tests were used to assess the significance of the observed differences. **** *p* < 0.0001, ** *p* < 0.01, * *p* < 0.05, ns: non-significant.

The pericentric distribution of mH2A1 in mouse senescent cells characterized by the proximity of DNA damage (γH2AX foci) led us to investigate whether DNA damage within pericentric regions could trigger mH2A1 recruitment. Expression of the Cas9 nuclease fused to GFP (Cas9-GFP) together with a guide RNA (gRNA) targeting major satellite (MajS) repeats of pericentric regions in mouse cells allowed us to efficiently and specifically induce DSBs at pericentric heterochromatin (Figure 2). The efficiency of generating DSBs with this synthetic system was rigorously evaluated using DSB markers such as γH2AX and DNA repair protein and DNA damage response (DDR) markers [38]. Cas9-GFP formed nuclear foci, which co-localized with γH2AX-stained chromocenters (Figure 2A). mH2A1 specifically and massively associated with chromocenters upon DSB induction at pericentromeric sequences, but not when induced at minor satellites, (MinS) nor at telomeres (Telo) [39]. To assess the nature and extent of chromatin alterations occurring after DSB induction at pericentric regions, we measured the area and the average intensity of chromocenters, γH2AX, H3K9me3, HP1α and whole nucleus (Figure 2B). We observed that the size of chromocenters was increased upon DSB induction, while the average intensity of Hoechst-labeled DNA decreased. These results demonstrate that DSB-containing chromocenters are partially expanded as compared to control chromocenters. Chromatin relaxation was accompanied neither by the eviction of HP1α nor by a reduction in H3K9me3 (Figure 2B and Figure S1A,B). We thus asked if mH2A1 recruitment to pericentromeric regions was caused by DNA breakage or by the ensuing DNA repair process. To that end, we tested if treating cells with an ATM inhibitor (ATMi) (Ku55933, 20 μM) during DSB induction affected mH2A1 association. At 24 h post-transfection of Cas9-gRNA majS, the number of mH2A1-positive cells (corresponding to cells presenting pronounced pericentric mH2A1 staining) decreased in ATMi-treated cells (23% ± 5 SD vs. 51% ± 8 SD) (Figure S2A), suggesting that mH2A1 recruitment is in part dependent on ATM activation. We next assessed the role of HIRA, a chaperone necessary for the recruitment of mH2A1 to SAHFs [14] and required for chromatin reassembly after DSB repair [40] in the recruitment of mH2A1 to pericentric regions. Partial depletion of HIRA by siRNA (Figure S2B) reduced mH2A1 binding to pericentric regions upon DSB induction (40% ± 16 SD vs. 71% ± 13 SD) (Figure S2C,D). Our results indicate that mH2A1 recruitment to damaged pericentric regions requires a functional repair pathway.

### 3.3. mH2A1 Recruitment to Pericentric Regions Is Not Cell Type-Dependent

To assess whether mH2A1 association with pericentric regions upon DSBs is cell type-specific, we induced senescence in human MCF-7 breast cancer cells using etoposide. Using the same protocol as for murine fibroblast, we confirmed senescence induction using SA-βgal marker (58% ± 5.4 SD of SA-βgal-positive cells) and we quantified the expression of some relevant mRNAs through quantitative real-time PCR (qPCR) known to be upregulated upon senescence induction (Figure S3A,B) [41]. As for mouse cells, in addition to a general diffuse staining throughout the nucleus, the most striking feature of mH2A1 distribution was a pronounced accumulation at dense HP1α -stained foci (Figure S3C). The fraction of senescent cells with pericentric mH2A1 staining was ∼70% ± 19 SD of cells, equivalent to the number of SA-βgal-positive cells (Figure S3A). A fraction of proliferative cells also exhibited pronounced pericentric mH2A1 staining (∼12.5% ± 6 SD cells) (Figure S3C), a similar proportion as SA-βgal-positive cells observed in proliferative conditions (∼12.7% ± 1.2 SD) (Figure S3A). Total mH2A1 protein levels were unchanged in senescent cells compared to proliferative cells (Figure S3D), suggesting that the native human mH2A1 content was redistributed to pericentric regions upon senescence induction. Surprisingly, despite an increase in γH2AX protein levels in senescent cells (Figure S3D,E), γH2AX foci were not associated with pericentric regions.

### 3.4. DSBs Are Not Necessary for mH2A1 Recruitment to Pericentric Regions

**Figure 3:**
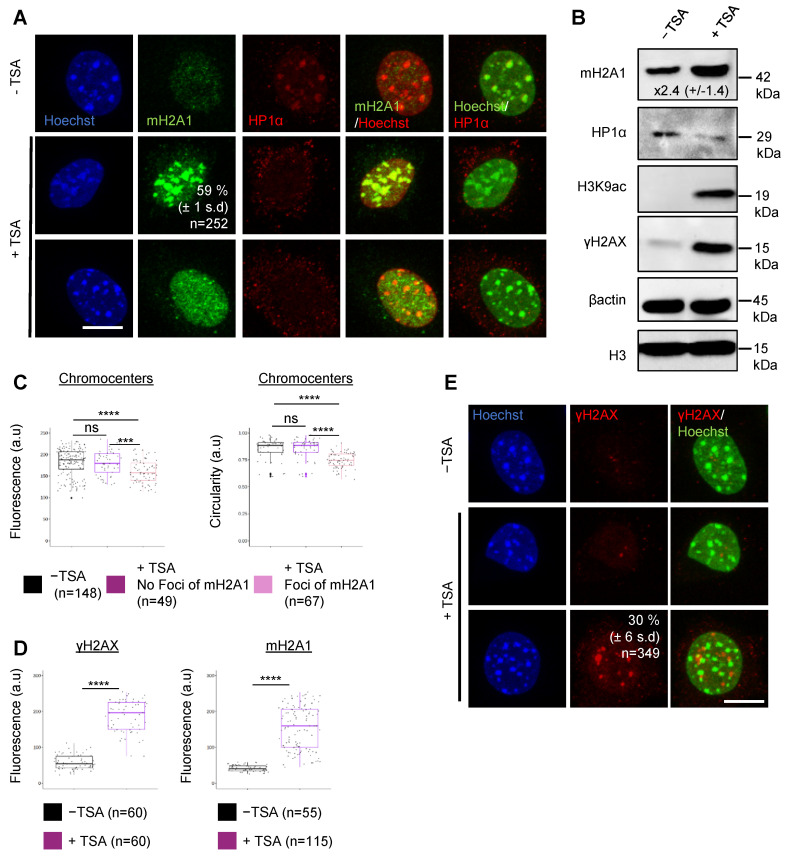
TSA treatment promotes mH2A1 recruitment to pericentric regions in the absence of satellite DSBs. (**A**) IF confocal images of untreated cells or cells treated with 500 nM of TSA over 48 h, stained with Hoechst and antibodies specific for mH2A1 and HP1α. Percentage of cells presenting mH2A1 foci at chromocenters is shown, represented as means ± SD from 3 biological replicates. Scale bar = 10 μm. (**B**) Immunoblots of mH2A1, HP1α, H3K9ac, γH2AX, and β-actin in protein extracts prepared from untreated and TSA-treated cells. Apparent molecular weights are indicated. Fold change increase in mH2A1 protein levels, normalized by β-actin from 4 biological replicates (as mean ± SD), is indicated. (**C**) Quantifications of the mean chromocenters (Hoechst-dense labelling) fluorescence and circularity in untreated and TSA-treated cells, taken from 2 biological replicates. TSA-treated cells are divided in two groups, depending on the presence of mH2A1 foci. (**D**) Quantifications of the mean γH2AX and mH2A1 fluorescence intensities in untreated and TSA-treated cells, taken from 2 biological replicates. The number of cells analyzed for each condition is given (n). (**E**) Same as in (**A**) but with γH2AX labelling. Percentage of cells presenting γH2AX foci at chromocenters is shown, represented as mean ± SD from 3 biological replicates. Scale bar = 10 μm. On boxplots, each point corresponds to the mean number of foci per cell. Wilcoxon tests were used to assess the significance of the observed differences. **** *p* < 0.0001, *** *p* < 0.001, ns: non-significant.

We then investigated if the pericentric recruitment of mH2A1 only occurred upon DNA damage in murine fibroblasts. To that end, we treated murine fibroblast L929 cells with TSA (500 nM, 48 h), an HDAC inhibitor previously shown to promote mH2A1 recruitment to pericentromeres in human cells [17] (Figure 3). We found that mH2A1 associated with pericentromeres in 59% (±1 SD) of TSA-treated cells, while HP1α was evicted from pericentric regions in all TSA-treated cells (Figure 3A). The significant increase in mH2A1 protein levels upon TSA treatment likely promoted this recruitment (Figure 3B). To assess the nature and extent of TSA-induced chromatin alterations at chromocenters, we measured nuclear fluorescence intensity and circularity in control and TSA-treated cells, separating TSA-treated cells without mH2A1 foci from TSA-treated cells with mH2A1 foci (Figure 3C). Interestingly, we found that fluorescence intensity and circularity of Hoechst-labelled chromocenters of TSA-treated cells with mH2A1 foci were reduced compared to control cells and TSA-treated cells without mH2A1 foci. We conclude that hyperacetylation leads to partial decondensation and expansion of mouse chromocenters, which incorporate mH2A1. Although γH2AX phosphorylation levels (Figure 3B,D) and the overall number of γH2AX foci (∼30% ± 1 SD) and γH2AX-foci-positive cells (Figure 3E) increased in TSA-treated cells, γH2AX foci did not colocalize with chromocenters. Thus, TSA treatment promoted mH2A1 relocalization to undamaged chromocenters. These results highly suggest that presence of DSBs is not necessary for mH2A1 recruitment to pericentric regions.

### 3.5. Recruitment of mH2A1 Proteins to Pericentric Heterochromatin Depends on Chromocenter Partial Decondensation

**Figure 4:**
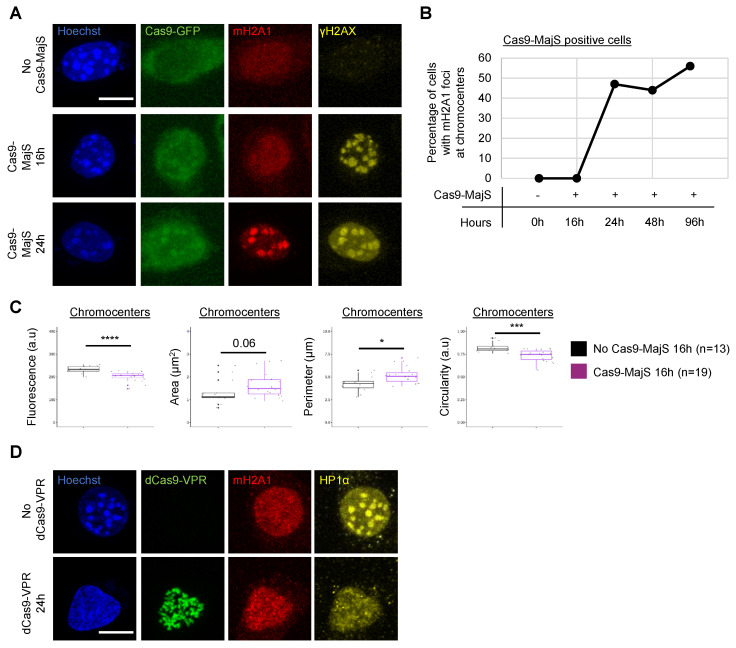
mH2A1 recruitment to DSBs correlates with chromocenter decondensation. (**A**) IF confocal images of 0 h-, 16 h-, or 24 h-transfected cells co-expressing Cas9-GFP and MajS-gRNAs, stained with Hoechst and antibodies specific for mH2A1 and γH2AX. Scale bar = 10 μm. (**B**) Line plot showing the percentage of cells with mH2A1 foci at chromocenters after different time points of Cas9-MajS transfection (0 h, 16 h, 24 h, 48 h, and 96 h). Percentages are based on Cas9-MajS-positive cells. One biological experiment was performed for each time point. (**C**) Quantifications of the mean fluorescence, area, perimeter, and circularity of chromocenters (Hoechst-dense labeling) after 16 h of transfection without (no Cas9-MajS 16 h) and/or with MajS gRNA (Cas9-MajS 16 h). The number of cells analyzed for each condition is given (n). Each point corresponds to the mean number of foci per cell. Wilcoxon tests were used to assess the significance of the observed differences. * *p* < 0.05, *** *p* < 0.001, **** *p* < 0.0001. (**D**) IF confocal images of 48 h post-transfected cells co-expressing dCas9-VPR and MajS gRNA or not, stained with Hoechst and antibodies specific for mH2A1 and HP1α. Scale bar = 10 μm.

The only parameter that seemed to be shared by the different insults previously tested is decondensation of the chromocenters. So, we investigated whether mH2A1 recruitment was a consequence or a cause of chromocenter decondensation. To that end, we induced Cas9-MajS DBS for varying durations (16 h, 24 h, 48 h, and 96 h) and measured mH2A1 recruitment relative to the extent of chromocenter decondensation. We show that mH2A1 was recruited only after 24 h of transfection (Figure 4A,B) even though chromocenters were already decondensed after 16 h of transfection (Figure 4C). As a means of generalizing these observations, we treated cells for varying times with TSA (0 h, 24 h, 48 h of TSA treatment). After 24 h of treatment, we observed that mH2A1 formed foci at chromocenters in 30% of cells, while 70% of cells already presented decondensed chromocenters (Figure S4A,B). mH2A1 foci formation at chromocenters increased progressively over the duration of the treatment and correlated with the decrease in fluorescence intensity (Figure S4C). These results suggest that mH2A1 recruitment appears as a consequence of chromocenter decondensation.

Finally, we transfected cells using nuclease-null Cas9 (dCas9) fused with a tripartite activator (VP64-p65-Rta (VPR)) and a gRNA targeting MajS, known to induce chromocenter decondensation [42,43]. The loss of HP1α foci combined with a disappearance of the Hoechst chromocenter staining pattern suggested that the decondensation of chromocenters was more important than in our previous experiments (Figure 4D). Under this condition, we never detected the presence of mH2A1 foci at chromocenters. We conclude that only “partial” decondensation of chromocenters promotes mH2A1 recruitment.

### 3.6. The “H2A-like” Domain of mH2A1 Is Sufficient to Recruit mH2A1 Proteins to Pericentric Heterochromatin

To test if pericentric recruitment of mH2A1 was isoform-specific, we co-transfected cells with either a plasmid coding for Flag-mH2A1.1 or a plasmid coding for Flag-mH2A1.2 (Figure S5A) in addition to the plasmids expressing Cas9-GFP/MajS gRNA. We observed that both isoforms were recruited to pericentric regions upon DSBs (Figure S5B). Similar results were obtained in TSA-treated cells (Figure S5C). To investigate which protein domains of mH2A1 are necessary for its recruitment to pericentric chromatin, we generated GFP fusions with mH2A1.1 (WT) and with truncation mutants that eliminate either its macrodomain (ΔM) or its macrodomain and linker (ΔLM) (Figure S6A,B). Transient expression of these GFP fusions in TSA-treated cells showed that all mH2A1.1-truncated forms were recruited to pericentric regions (Figure S6C). In contrast, diffuse faint staining of H2B-GFP and GFP alone was seen throughout the nucleus with no preference for pericentric regions (Figure S6C).

### 3.7. mH2A1 Is Not Required for Pericentric Heterochromatin Organization

**Figure 5:**
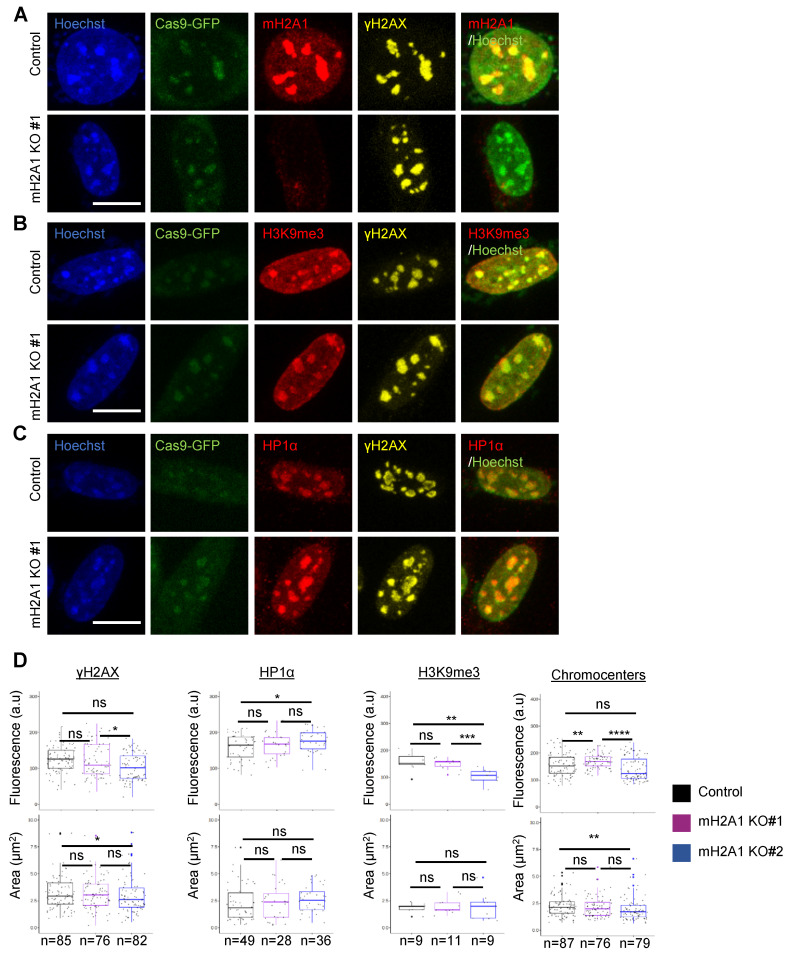
DSB-induced chromocenter relaxation is independent of mH2A1. (**A**) IF confocal images of cells co-expressing Cas9-GFP and MajS gRNA, stained with Hoechst and antibodies specific for mH2A1 and γH2AX in control and mH2A1 KO #1 cells. Scale bar = 10 μm. (**B**) As in (**A**) but cells were stained with Hoechst and antibodies specific for H3K9me3 and γH2AX. (**C**) As in (**A**) but cells were stained with Hoechst and antibodies specific for HP1α and γH2AX. (**D**) Quantifications of the mean areas and fluorescence intensities of γH2AX, H3K9me3, HP1α and chromocenters (Hoechst-dense labelling) in control and mH2A1 KO clones, taken from > 3 biological replicates, except for HP1α and H3K9me3 (only 1 biological replicate). The number of cells analyzed for each condition is given (n). Each point corresponds to the mean number of foci per cell. Wilcoxon tests were used to assess the significance of the observed differences. **** *p* < 0.0001, *** *p* < 0.001, ** *p* < 0.01, * *p* < 0.05, ns: non-significant.

We generated mH2A1 KO L929 mouse cell lines using a CRISPR/Cas9 system. We selected two mH2A1 KO clones (mH2A1 KO #1 and mH2A1 KO #2) showing no expression of mH2A1 protein by immunofluorescence visualization and immunoblot analysis (Figure 5A and Figure S7). Upon DSB induction using Cas9-GFP/MajS gRNA, we still observed γH2AX, HP1α and H3K9me3 foci decorating pericentric regions in mH2A1 KO clones (Figure 5A–C). Mean average intensity and area of γH2AX, HP1α and H3K9me3 foci showed no significant differences between WT and either one of the mH2A1 KO clones (Figure 5D). Partial decondensation of chromocenters induced by DSBs also occurred in mH2A1 KO cells. Also, the percentages of cells expressing SA-βgal after etoposide treatment, as well as γH2AX, HP1α, H3K9me3 labelling and chromocenter organization upon etoposide-induced senescence and TSA treatment, were similar in WT and mH2A1 KO cells (Figures S8 and S9).

### 3.8. mH2A1 Regulates Pericentromeric RNA Transcription

**Figure 6:**
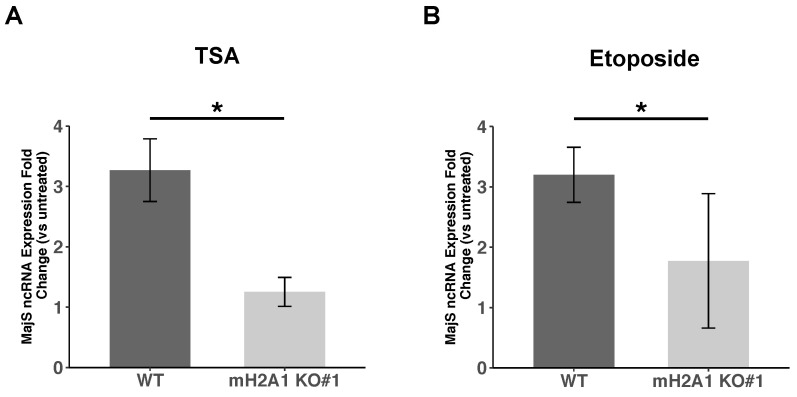
mH2A1 is necessary for pericentric ncRNA upregulation upon chromocenter decondensation. Reverse transcription quantitative PCR (RT-qPCR) with total RNA isolated from unsynchronized WT or macroH2A1 KO#1 mouse L919 cells. (**A**) The ratio of major satellite repeat expression between cells treated with 500 nM TSA for 48 h and control cells are plotted. The data represent the mean ± SD of four independent experiments. (**B**) The ratio of major satellite repeat expression between cells treated with 12.5 μM etoposide for 24 h and control cells is plotted. The data represent the mean ± SD of three independent experiments. For (**A**,**B**), amplified signals were normalized to GAPDH. Wilcoxon tests were used to assess the significance of the observed differences. (* *p* = 0.02857 for (**A**), *p* = 0.02597 for (**B**)).

We finally asked whether mH2A1 could regulate pericentromeric satellite repeat transcription (MajS ncRNA) in cells exposed to previously used treatments. In WT cells, we observed a 3- to 5-fold increase in the transcriptional level of MajS ncRNA after TSA treatment or etoposide-induced senescence, respectively (Figure 6). Strikingly, this transcriptional upregulation of MajS ncRNA was largely compromised in mH2A1 KO cells following the same treatments. This finding highlights a key role of mH2A1 in upregulating the transcriptional expression or stabilization of MajS ncRNA under conditions of cellular stress.

## 4. Discussion

In this study, we demonstrate that the histone variant mH2A1 massively associates with partially decondensed pericentric regions upon stress. Our findings further show that mH2A1 contributes to the adaptation of transcriptional expression of pericentric noncoding RNA, without affecting the establishment and maintenance of pericentric chromatin reorganization. We discovered that perturbation of pericentric heterochromatin led to significant enrichment in mH2A1 at these regions. Moreover, partial decondensation of pericentric chromatin was a prerequisite for mH2A1 recruitment, while complete decondensation of pericentric chromatin prevented mH2A1 association. Our results illustrate that mH2A1 recruitment to decondensed pericentric chromatin occurs independently of the source of damage and may play a general role in the response to genotoxic stress. The finding that mH2A1 is recruited to partially decondensed pericentric regions was unexpected, as previous studies had mainly associated this variant with highly condensed regions, such as the Xi chromosome [17,19,20,21,22] and SAHFs [14]. We also show that relaxation of pericentric chromatin upon insults occurred in cells in which mH2A1 was knocked out. Therefore, the presence of mH2A1 does not seem to be necessary for decondensation to occur nor to be maintained, at least in the context of long-term external insults or stress. We can not exclude, however, that other variants compensate for the absence of mH2A1 [12]. Our observations are nonetheless consistent with previous studies showing that mH2A1 is not involved in condensation of the Xi chromosome [23,44,45]. Murine cells do not form SAHFs during senescence [46], making it difficult to identify senescent states. Hence, the presence of mH2A1 at pericentric regions could be used as a marker of DSB-induced senescent mouse cells. In addition, we demonstrate that mH2A1 is recruited to pericentric regions in etoposide-induced human senescent MCF-7 cells that do not form SAHFs. Further research is required to confirm whether mH2A1 could serve as a universal marker for senescent cells, including replicative- and oncogenic-induced senescent cells [47]. Since decondensation of pericentric chromatin has been proposed to be a new marker of senescence [48], mH2A1 could be a promising alternative for the identification of senescent cells, particularly in cases where SAHFs are absent or not easily detectable. mH2A1 was massively recruited to pericentric regions in response to DSB induction. Previous research has established that mH2A1 plays a significant role in DSB repair efficiency, acting on the two main repair pathways, Homologous Recombination (HR) and Non-Homologous End-Joining (NHEJ) [49,50,51,52,53,54]. Although, in our study, we did not investigate the role of mH2A1 in the repair of pericentric DSBs because the CRISPR Cas9 system used leads to permanent DSBs (Cas9 is always active), the phosphorylation intensity of γH2AX remains consistent in both WT and KO conditions. Considering that γH2AX phosphorylation occurs through the involvement of ATM, it is conceivable that the recruitment of ATM to DNA double-strand breaks (DSBs) is not influenced by mH2A1. To further investigate the role of mH2A1 in the repair of pericentric DSBs, the use of a degradable Cas9 [38] could be considered. The role of mH2A1 in DSB repair, depending on the chromatin landscapes, particularly in heterochromatin, had not been thoroughly examined. Our results suggest that mH2A1 is massively recruited to DSBs present on constitutive heterochromatin (pericentric regions), whereas it did not accumulate at DSBs present on heterochromatin with nucleosomes that lacked a phased configuration, such as centromeric (MinS) DSBs, or DSBs associated with specific chromatin, such as telomeric DSBs. These results suggest that the recruitment of mH2A1 may be influenced by the pre-existing chromatin status of the damaged region. Grigoryev et al. proposed that mH2A1 replaces HP1α in pericentric regions to maintain their condensation state, based on an inverse correlation between the recruitment of mH2A1 and loss of HP1α in lymphocytes [17]. We found that mH2A1 can be recruited to pericentric regions independently of HP1α. Moreover, we did not observe retention of HP1α in mH2A1 KO cells upon TSA treatment. Therefore, our results indicate that the model may not apply universally and may be specific to certain cell types or conditions. Our results suggest that the two proteins may have distinct functions at pericentric regions. Under normal physiological conditions, the levels of MajS ncRNA are very low [55,56], but in various pathological conditions, such as cellular stress [57] and cancer [58], the levels are significantly increased. Here, we found that in mH2A1 KO cells, expression of MajS ncRNA was no longer stimulated upon TSA and etoposide treatment. While the specific function of MajS ncRNA in the stress response is unclear, it has been shown in S. pombe and in plants that these transcripts could participate in the re-establishment of heterochromatin [59]. Moreover, depletion of MajS ncRNA in mouse cells during early developmental stages has been shown to result in the failure of pericentric chromatin reorganization into chromocenters [56]. Therefore, it is possible that mH2A1 plays a role in ensuring proper expression of MajS ncRNA to restore pericentric chromatin homeostasis after stress. Based on these findings, it would be valuable to conduct additional research to elucidate the precise mechanisms by which mH2A1 modulates the expression of MajS ncRNA and its possible contribution to maintaining chromatin stability in response to stress.

## Supplementary Materials

The following supporting information can be downloaded at: https://www.mdpi.com/article/10.3390/cells12172175/s1, Figure S1: Cas9-induced DSBs induction does not perturb HP1a and H3K9me3 at pericentric regions; Figure S2: ATM and the chaperone HIRA participate in the recruitment of mH2A1 to pericentric regions upon Cas9-induced DSBs; Figure S3: mH2A1 is recruited to pericentric regions in senescent human MCF-7 cells; Figure S4: The recruitment of mH2A1 to chromocenters upon TSA treatments is proportional to the decondensation states of chromocenters; Figure S5: Both isoforms of the histone variant mH2A1 are recruited to pericentric heterochromatin upon DSBs and TSA treatment; Figure S6: The « H2A-like » domain of mH2A1 is sufficient for its recruitment to pericentric regions; Figure S7: Validation of mH2A1 KO clones using immunoblot analysis; Figure S8: Chromocenter organization in senescent cells is independent of mH2A1; Figure S9: Chromocenter organization in TSA-treated cells in independent of mH2A1.

## Appendix A

**Table A1:** gRNAs.

gRNAs	gRNA Sequence	PAM
gRNA 1	ATTCGGCAACACGCCCCCGC	TGG
gRNA 2	CACGCCTCCGCCGGCCAAAA	AGG

**Table A2:** Plasmids *.

Cas9	pSpCas9(BB)-2A-Puro (PX459) V2.0 (Plasmid #62988, addgene)
Cas9-GFP	pSpCas9(BB)-2A-GFP (PX458) (Plasmid #48138, addgene)
Cas$9_{g}RNA1$	pSpCas9(BB)-2A-Puro gRNA 1 ex4 H2AFY MS
Cas9-GF$P_{g}RNA2$	pSpCas9(BB)-2A-GFP (PX458) gRNA 2 ex4 H2AFY Ms
MajS gRNA	pEX-A-U6-MaSgRN$A_{P}uro_{R}$ (Plasmid #84780, addgene)
MinS gRNA	pEX-A-U6-MiSgRN$A_{P}uro_{R}$ (Plasmid #84781, addgene)
Telo gRNA	pEX-A-U6-TelgRN$A_{P}uro_{R}$ (Plasmid #84782, addgene)
EGFP	pLVX-EGFP
EGFP-mH2A1.1	pLVX-EGFP-mH2A1.1
EGFP-mH2A1 delMD (aa1-179)	pLVX-EGFP-mH2A1 delMD (aa1-179)
EGFP-mH2A1 delLMD (aa1-123)	pLVX-EGFP-mH2A1 delLMD (aa1-123)
H2B-EGFP	pBOS-H2B-GFP (BD Pharmingen)
Flag-mH2A1.1	Given by Marcus Buschbeck
Flag-mH2A1.2	Given by Marcus Buschbeck
dCas9-VPR	Given by Fabian Erdel [43]

* Plasmids and sequences available upon request.

**Table A3:** siRNA.

siRNA	Sequence
HIRA (5’ -> 3’)	GGAGAUGACAAACUGAUUAUU

**Table A4:** Primers.

Target	Forward Primer	Reverse Primer
18S mRNA	CCCTATCAACTTTCGATGGTAGTCG	CCAATGGATCCTCGTTAAAGGATTT
ANKRD1	AGTAGAGGAACTGGTCACTGG	TGGGCTAGAAGTGTCTTCAGAT
CDKN1A (p21)	GACACCACTGGAGGGTGACT	CAGGTCCACATGGTCTTCCT
CXCL1	GAAAGCTTGCCTCAATCCTG	CACCAGTGAGCTTCCTCCTC
EDN1	CAGCAGTCTTAGGCGCTGAG	ACTCTTTATCCATCAGGGACGAG
IL6	CCGGGAACGAAAGAGAAGCT	GCGCTTGTGGAGAAGGAGTT
IL8	CTTTCCACCCCAAATTTATCAAAG	CAGACAGAGCTCTCTTCCATCAGA
MajS	GACGACTTGAAAAATGACGAAATC	CATATTCCAGGTCCTTCAGTGTGC
GAPDH	AACTTTGGCATTGTGGAAGG	ACACATTGGGGGTAGGAACA
gRNA 1	CACCGATTCGGCAACACGCCCCCGC	AAACGCGGGGGCGTGTTGCCGAAT
gRNA 2	CACCGCACGCCTCCGCCGGCCAAAA	AAACTTTTGGCCGGCGGAGGCGTG

**Table A5:** Antibodies.

Antibody	Supplier (Reference)	Dilution (Use) *
HIRA	Cell signaling (D2A5E)	1/1000 (WB)
γH2AX	Abcam Ab26350 [9F3]	1/1000 (WB & IF)
γH2AX	Abcam Ab81299	1/1000 (IF)
mH2A1.2	Millipore #MABE61 Clone 14GT	1/1000
mH2A1	Millipore #AbE215	1/1000 (WB & IF)
H3K9me3	Abcam Ab8898	1/1000 (IF)
H3K9me3	Abcam (Ab1991)	1/1000 (WB)
Flag	Sigma (F3165) M2	1/1000 (WB & IF)
βactin	Abcam (Ab8227)	1/1000 (WB)
HP1α	Upstate #05-689	1/1000 (WB & IF)
H3K9ac	Upstate #06-942	1/1000 (WB)
LaminB1	Abcam Ab16048	1/1000 (WB)
GFP	Roche 1814460001	1/1000 (WB)
mH2A1-Nter	Abcam (Ab137117)	1/1000 (IF)
mH2A1.1	Ab αmH2A1.1 Home-made	1/1000 (IF)
Anti-mouse-Peroxidase	Sigma A2304	1/10.000 (WB)
Anti-Rabbit-Peroxidase	Sigma A0545	1/10.000 (WB)
Alexa Fluor 488 Anti-mouse	Invitrogen A11029	1/1000 (IF)
Alexa Fluor 647 Anti-Rabbit	Invitrogen A21245	1/1000 (IF)

* WB: Western Blot; IF: Immunofluorescence.
