# Supplementary material for: Recruitment of the Histone Variant MacroH2A1 to the Pericentric Region Occurs upon Chromatin Relaxation and Is Responsible for Major Satellite Transcriptional Regulation
Source: Cells. 2023 Aug 30;12(17):2175. doi: 10.3390/cells12172175 (PMC10486525; doi:10.3390/cells12172175)
Supplement: Supplementary file 1 [file cells-12-02175-s001.zip › Figure S1.pdf]

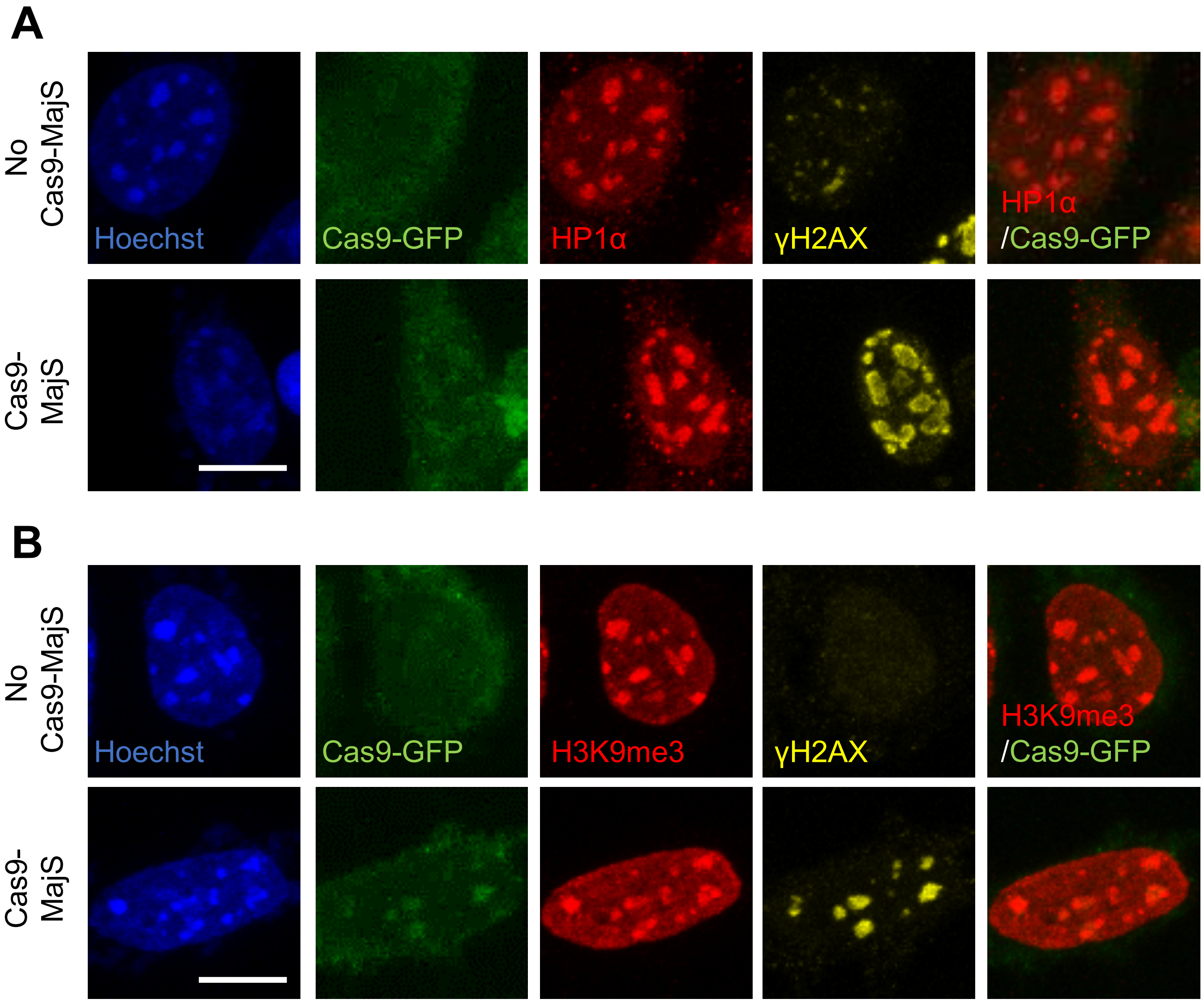

Figure S1. Cas9-induced DSBs induction does not perturb HP1α and H3K9me3 at pericentric regions. (**A**) IF confocal images of cells co-expressing Cas9-GFP and MajS gRNA or not, stained with Hoechst and antibodies specific for HP1α and γH2AX. (**B**) As in (A) but stained with antibodies specific for H3K9me3 and γH2AX. Scale bar = 10 μm.
