# Supplementary material for: Recruitment of the Histone Variant MacroH2A1 to the Pericentric Region Occurs upon Chromatin Relaxation and Is Responsible for Major Satellite Transcriptional Regulation
Source: Cells. 2023 Aug 30;12(17):2175. doi: 10.3390/cells12172175 (PMC10486525; doi:10.3390/cells12172175)
Supplement: Supplementary file 1 [file cells-12-02175-s001.zip › Figure S2.pdf]

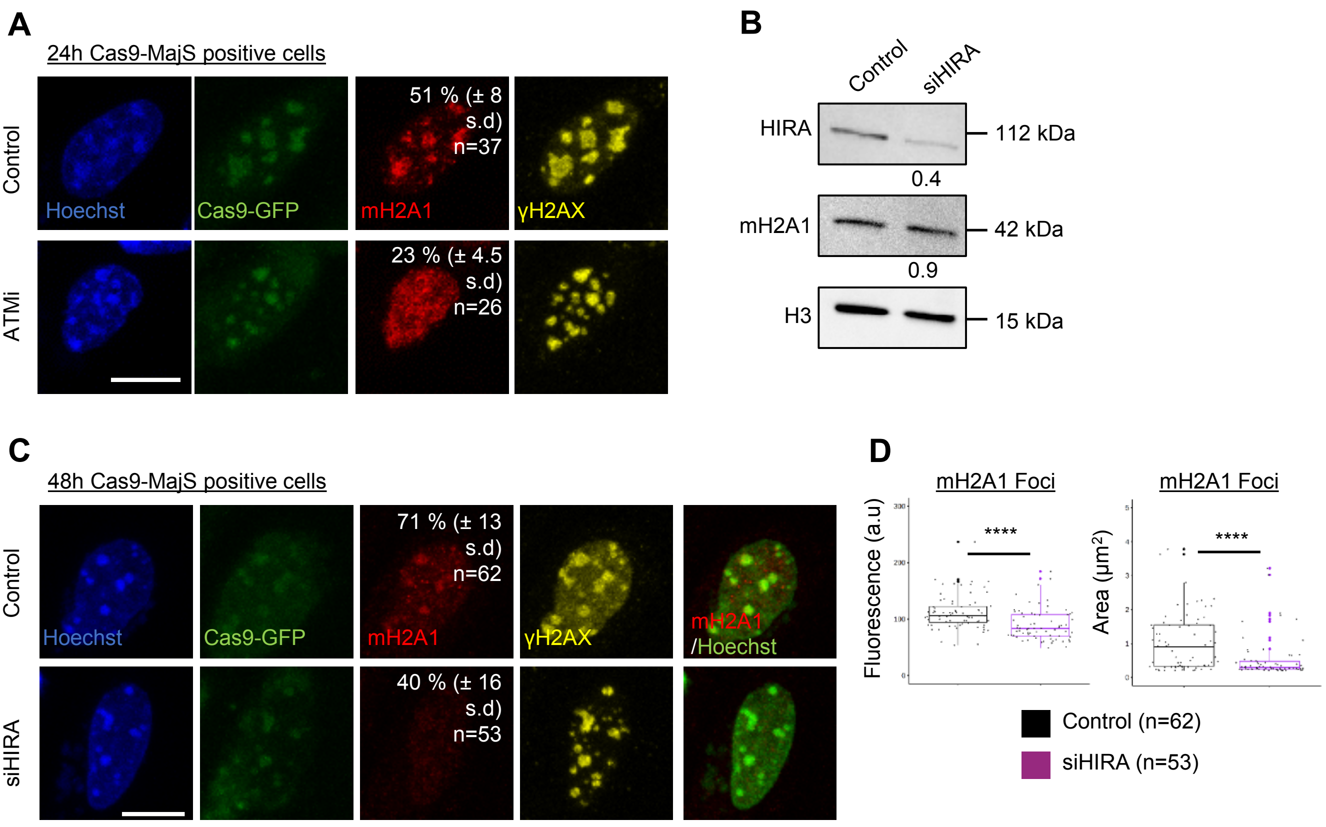

Figure S2. ATM and the chaperone HIRA participate in the recruitment of mH2A1 to pericentric regions upon Cas9-induced DSBs. (**A**) IF confocal images of cells co-expressing Cas9-GFP and MajS gRNA, stained with Hoechst and antibodies specific for mH2A1 and γH2AX in untreated and ATMi-treated cells (Ku55933, 20 µM). ATMi was added 1h before plasmid transfections. Cells were fixed 24h post-transfection. Percentage of cells presenting mH2A1 foci at MajS are shown, represented as means ± SD from 2 biological replicates. Scale bar = 10 µm. (**B**) Immunoblot analysis for HIRA, mH2A1 and H3 in protein extracts prepared from control and 72h post-transfected cells with an siRNA against HIRA. Apparent molecular weights are indicated. Quantifications of HIRA and mH2A1 protein expression are given, normalized by H3. (**C**) IF confocal images of 48h Cas9-MajS post-transfected cells co-expressing Cas9-GFP and MajS gRNA, stained with Hoechst and antibodies specific for mH2A1 and γH2AX in control and HIRA-partially depleted cells (siHIRA, 72 h post-transfection). Percentage of cells presenting mH2A1 foci at MajS are shown, represented as means ± SD from 2 biological replicates. Scale bar = 10 µm. (**D**) Quantifications of the mean fluorescence intensity and area of mH2A1 foci in control and HIRA partially depleted cells (siHIRA) in Cas9 positive cells, taken from 2 biological replicates. The number of cells analyzed for each condition is given (n). On boxplots, each point corresponds to the mean number of foci per cell. Wilcoxon tests were used to assess the significance of the observed differences. **** $P < 0.0001$.
