# Supplementary material for: Recruitment of the Histone Variant MacroH2A1 to the Pericentric Region Occurs upon Chromatin Relaxation and Is Responsible for Major Satellite Transcriptional Regulation
Source: Cells. 2023 Aug 30;12(17):2175. doi: 10.3390/cells12172175 (PMC10486525; doi:10.3390/cells12172175)
Supplement: Supplementary file 1 [file cells-12-02175-s001.zip › Figure S3.pdf]

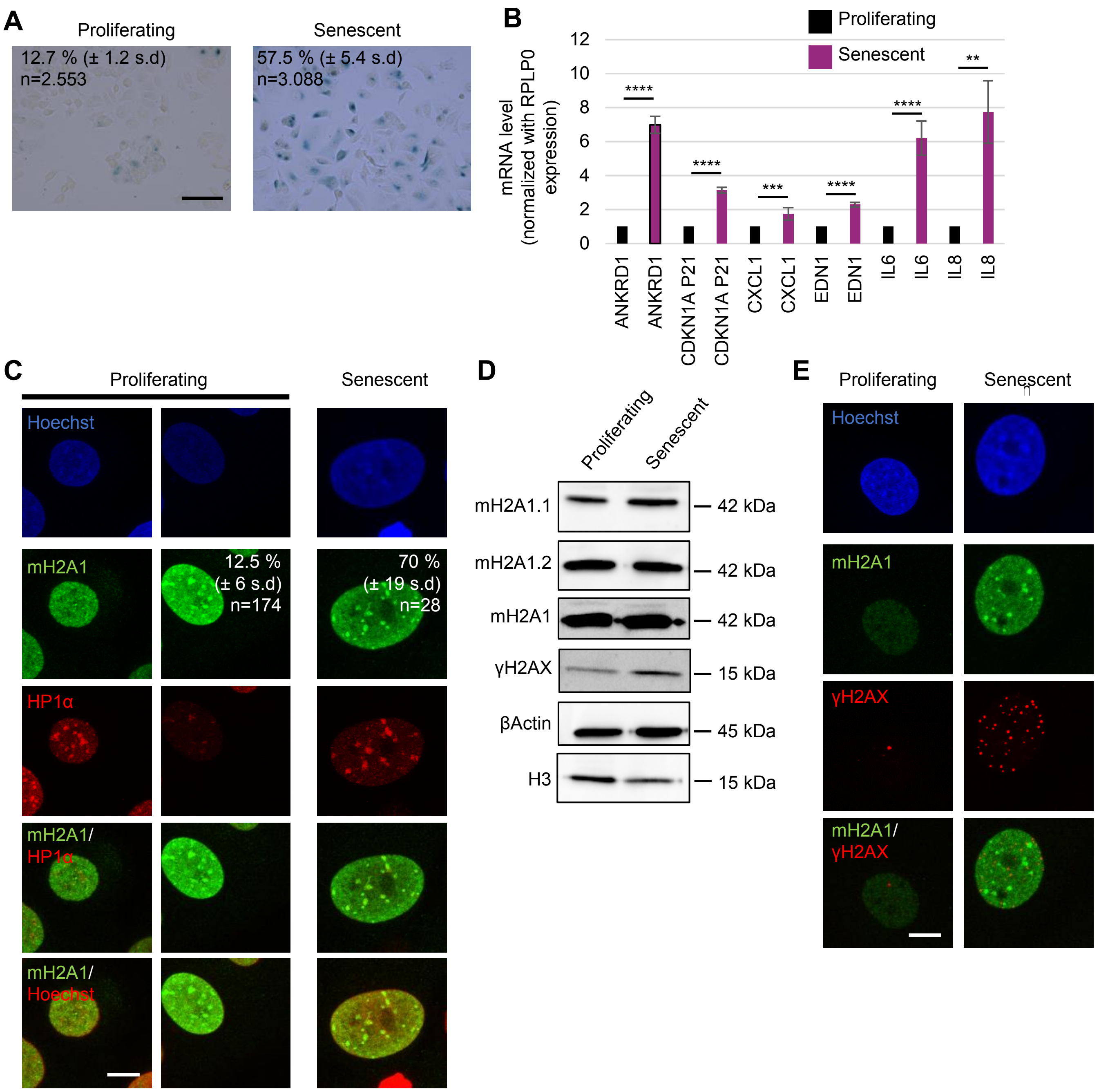


**Figure S3. mH2A1 is recruited to pericentric regions in senescent human MCF-7 cells.** (**A**) Representative images of a SA-βgal activity assessed by X-gal staining of proliferating and etoposide-treated (12.5 μM, 24h + 4 days release) senescent human MCF-7 cells. Percentage of SA-βgal-positive cells are given, represented as means ± SD from 2 biological replicates. The number of counted cells is given (n). (**B**) Relative expression of mRNA senescence markers using real-time PCR in proliferating and senescent MCF-7 cells. Assay was performed in a technical triplicate and normalized to 18S mRNA. The bars represent the mean ± SD. T-tests were used to assess the significance of the observed differences. **** $P < 0.0001$, *** $P < 0.001$**, $P < 0.01$. (**C**) IF confocal images of proliferating and senescent MCF-7 human cells stained with Hoechst and antibodies specific for mH2A1 and HP1α. Percentage of cells presenting mH2A1 foci at pericentric regions are shown, represented as means ± SD from two biological replicates. Scale bar = 10 μm. (**D**) Immunoblot analysis for mH2A1.1, mH2A1.2, mH2A1, γH2AX and βactin in protein extracts prepared from proliferating and senescent cells. Apparent molecular weights are indicated. (**E**) IF confocal images of proliferating and senescent MCF-7 cells stained with Hoechst and antibodies specific for mH2A1 and γH2AX. Scale bar = 10 μm.
