# Supplementary material for: Recruitment of the Histone Variant MacroH2A1 to the Pericentric Region Occurs upon Chromatin Relaxation and Is Responsible for Major Satellite Transcriptional Regulation
Source: Cells. 2023 Aug 30;12(17):2175. doi: 10.3390/cells12172175 (PMC10486525; doi:10.3390/cells12172175)
Supplement: Supplementary file 1 [file cells-12-02175-s001.zip › Figure S4.pdf]

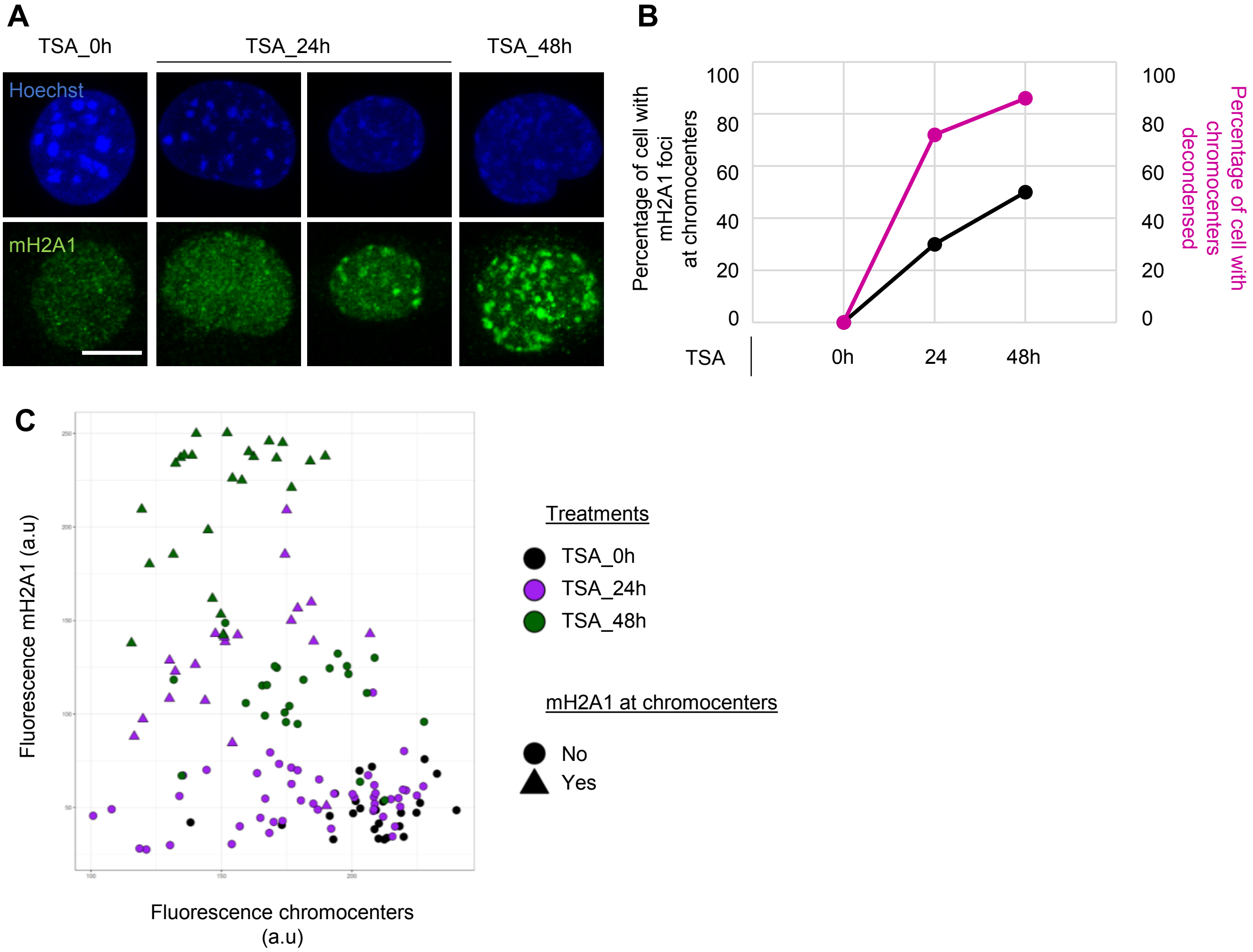

Figure S4. The recruitment of mH2A1 to chromocenters upon TSA treatments is proportional to the decondensation states of chromocenters. (**A**) IF confocal images of cells after 0h, 24h and 48h of TSA treatment, stained with Hoechst and antibody specific for mH2A1. Scale bar = 10 µm. (**B**) Lines plot showing the percentage of cells with mH2A1 foci at chromocenters and the percentage of cells with decondensed chromocenters at different time points TSA treatment (0h, 24h, and 48h). One biological experiment was performed for each time point. (**C**) Scatter plot showing the correlation between the fluorescence of chromocenters and the fluorescence of mH2A1 per cell at different durations of TSA treatment (0h, 24h and 48h). Each point corresponds to the mean number of foci per cell. Presence of mH2A1 foci at chromocenters is also indicated.
