# Supplementary material for: Recruitment of the Histone Variant MacroH2A1 to the Pericentric Region Occurs upon Chromatin Relaxation and Is Responsible for Major Satellite Transcriptional Regulation
Source: Cells. 2023 Aug 30;12(17):2175. doi: 10.3390/cells12172175 (PMC10486525; doi:10.3390/cells12172175)
Supplement: Supplementary file 1 [file cells-12-02175-s001.zip › Figure S5.pdf]

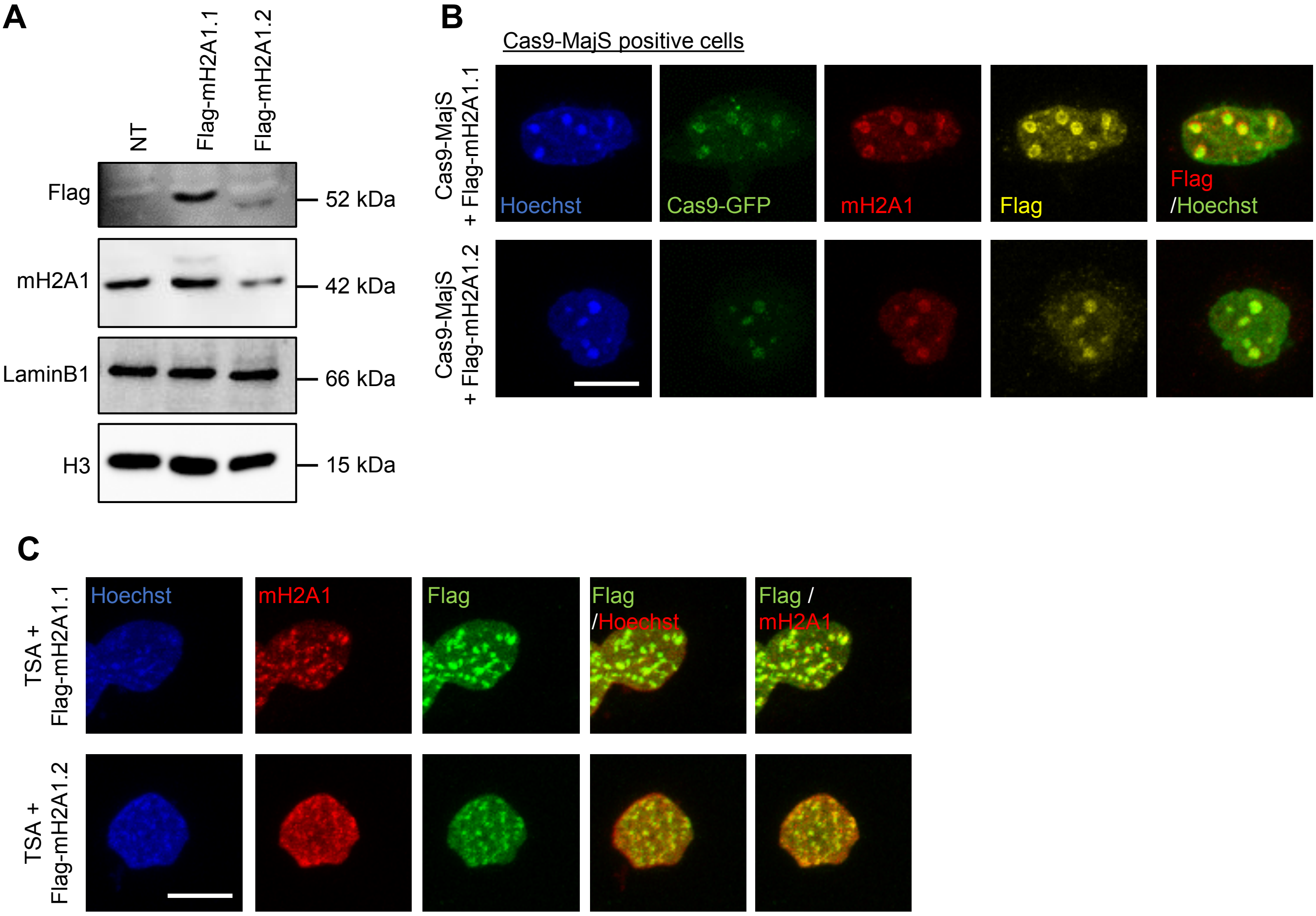


**Figure S5. Both isoforms of the histone variant mH2A1 are recruited to pericentric heterochromatin upon DSBs and TSA treatment.** (**A**) Immunoblot analysis for Flag, mH2A1, LaminB1 and H3 in extracts prepared from control and 48h post-transfected cells with plasmids coding for mH2A1.1-Flag (Flag-1.1) or mH2A1.2-Flag (Flag-1.2). Apparent molecular weights are indicated. (**B**) IF confocal images of cells co-expressing Cas9-GFP, MajS gRNA and mH2A1 Flag-tagged isoforms, stained with Hoechst and antibodies specific for Flag and mH2A1. Scale bar =10 μm. (**C**) IF confocal images of cells treated with 500 nM of TSA during 48h and transfected with mH2A1 Flag-tagged isoforms, stained with Hoechst and antibodies specific for Flag and mH2A1. Scale bar =10 μm.
