# Supplementary material for: Recruitment of the Histone Variant MacroH2A1 to the Pericentric Region Occurs upon Chromatin Relaxation and Is Responsible for Major Satellite Transcriptional Regulation
Source: Cells. 2023 Aug 30;12(17):2175. doi: 10.3390/cells12172175 (PMC10486525; doi:10.3390/cells12172175)
Supplement: Supplementary file 1 [file cells-12-02175-s001.zip › Figure S6.pdf]

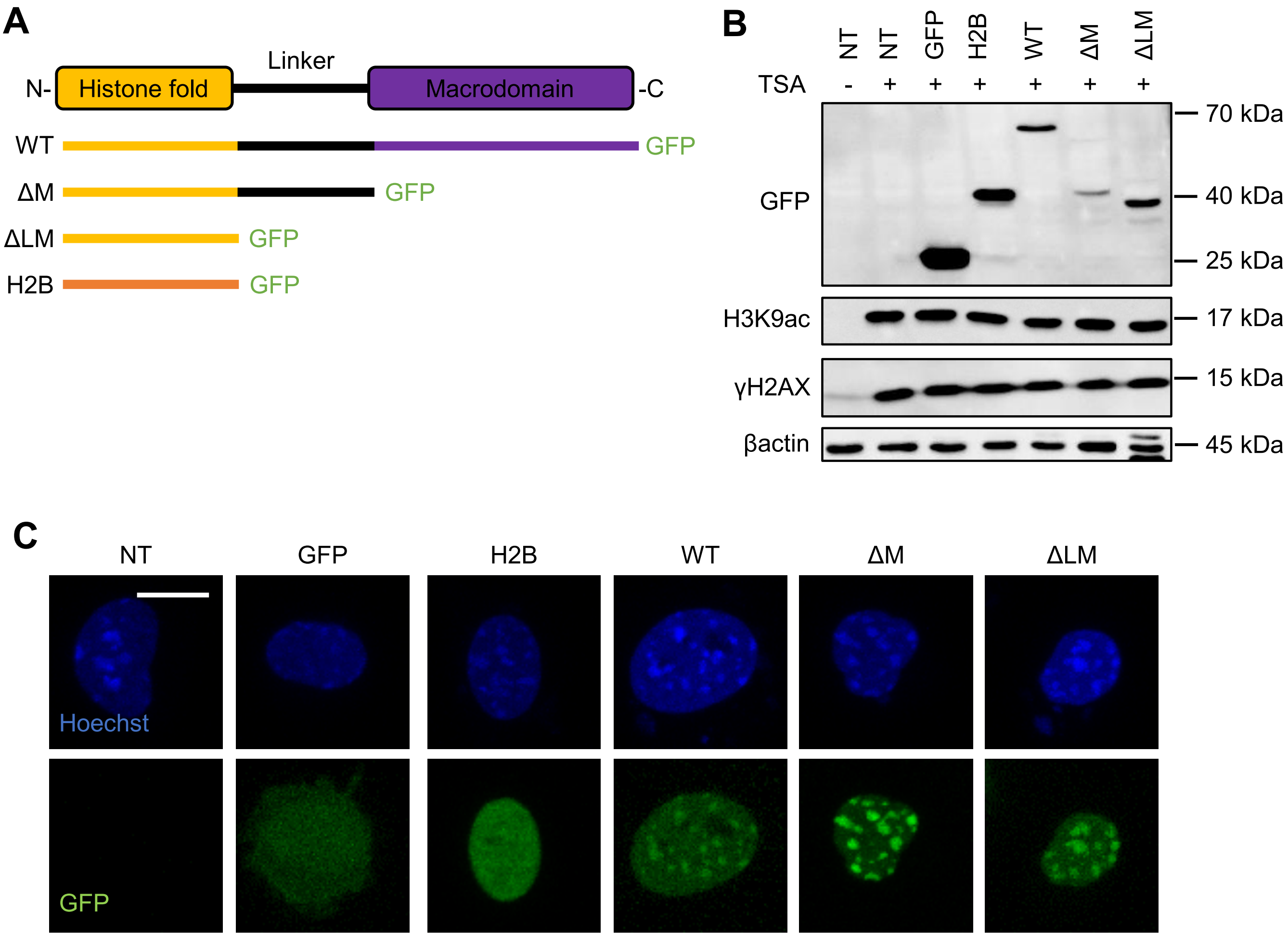

Figure S6. The « H2A-like » domain of mH2A1 is sufficient for its recruitment to pericentric regions. (**A**) Schema of mH2A1 variants used in the study: full-length wild type mH2A1.1-GFP (WT), constructs with deleted macro-domain (ΔM), constructs with depletion of both the linker and macro-domain (ΔLM) and H2B-GFP. (**B**) Immunoblot analysis for GFP, H3K9ac, γH2AX and β-actin in protein extracts prepared from TSA-treated cells (500 nM during 48h) co-transfected with mH2A1 variants, as indicated in (A). Apparent molecular weights are indicated. (**C**) IF confocal images of 48h TSA-treated cells and co-transfected with mH2A1 variants presented in (A), stained with Hoechst. Scale bar = 10 μm.
