# Supplementary material for: Recruitment of the Histone Variant MacroH2A1 to the Pericentric Region Occurs upon Chromatin Relaxation and Is Responsible for Major Satellite Transcriptional Regulation
Source: Cells. 2023 Aug 30;12(17):2175. doi: 10.3390/cells12172175 (PMC10486525; doi:10.3390/cells12172175)
Supplement: Supplementary file 1 [file cells-12-02175-s001.zip › Figure S7.pdf]

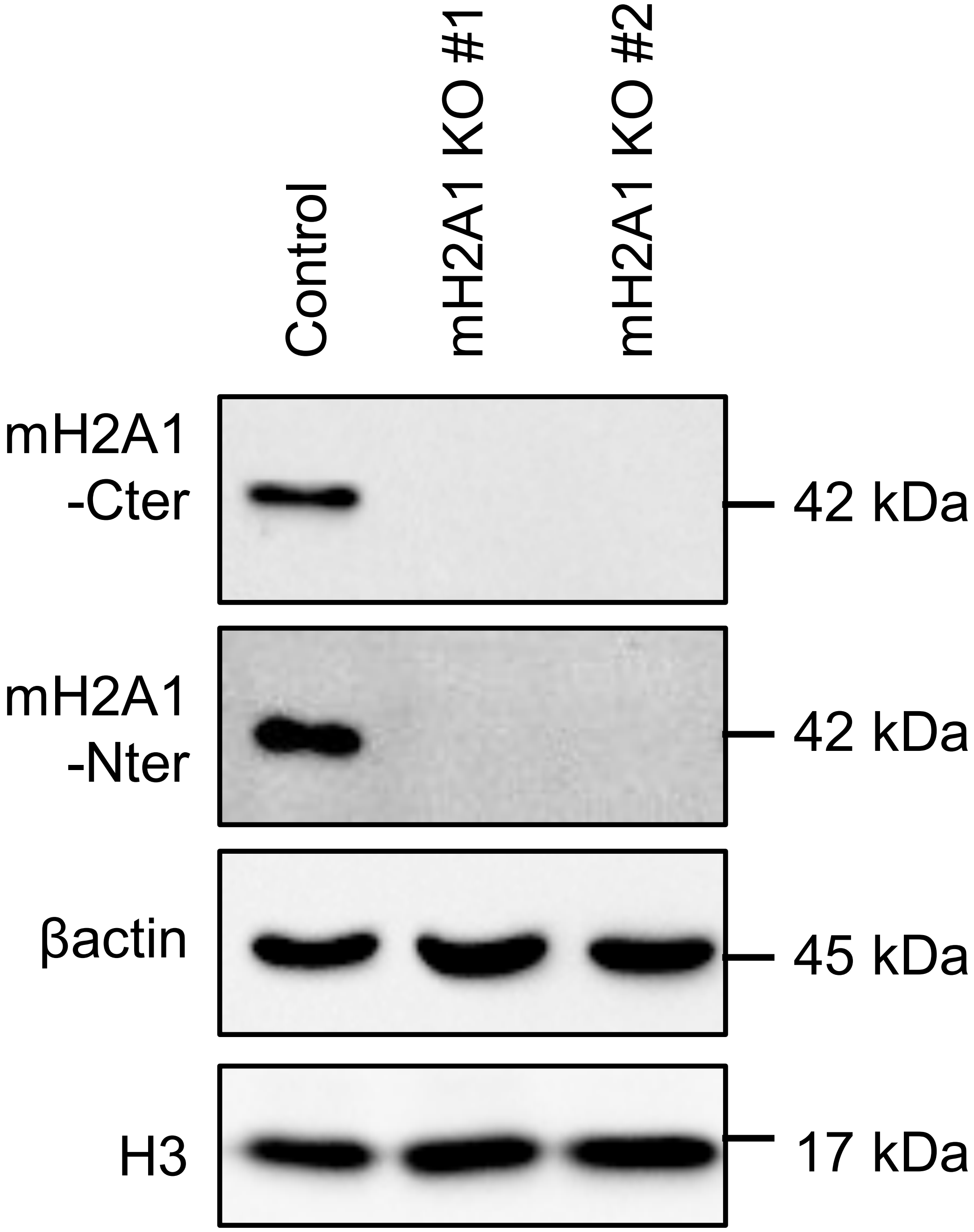

Figure S7. Validation of mH2A1 KO clones using immunoblot analysis. Immunoblot analysis of mH2A1, β-actin and H3 in protein extracts prepared from control and two mH2A1 KO clones (1, 2). To ensure the absence of mH2A1, two different mH2A1-specific antibodies were used, specific either of the C-terminal part (-Cter) or of the N-terminal part (-Nter) of mH2A1. Apparent molecular weights are indicated.
