# Supplementary material for: Recruitment of the Histone Variant MacroH2A1 to the Pericentric Region Occurs upon Chromatin Relaxation and Is Responsible for Major Satellite Transcriptional Regulation
Source: Cells. 2023 Aug 30;12(17):2175. doi: 10.3390/cells12172175 (PMC10486525; doi:10.3390/cells12172175)
Supplement: Supplementary file 1 [file cells-12-02175-s001.zip › Figure S8.pdf]

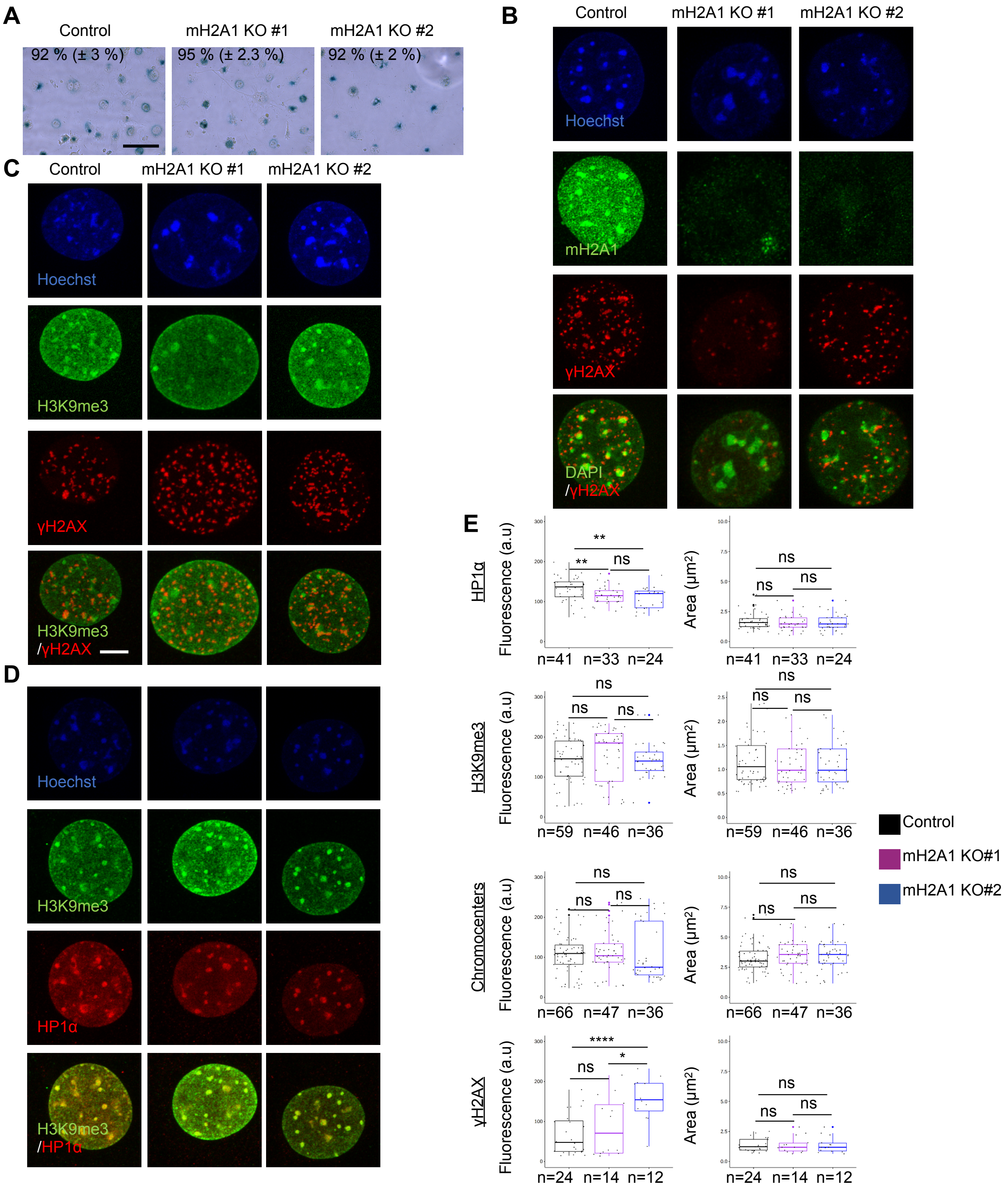

Figure S8. Chromocenter organization in senescent cells is independent of mH2A1. (**A**) Representative images of SA-βgal, assessed by X-gal staining for mouse control cell and the two mH2A1 KO clones upon senescence induction using etoposide treatment (12.5 μM, 24h and release during 4 days). Percentage of X-gal positive cells are given, represented as means ± SD from two biological replicates. Scale bar = 100 μm. (**B**) IF confocal images of senescent control cell and mH2A1 KO clones stained with Hoechst and antibodies specific for mH2A1 and γH2AX. Scale bar = 10 μm. (**C**) Same as in (B) but cells are stained with Hoechst and antibodies specific for H3K9me3 and γH2AX. (**D**) As in (B) but cells are stained with Hoechst and antibodies specific for H3K9me3 and HP1α. (**E**) Quantifications of HP1α, H3K9me3, chromocenters (Hoechst-dense labelling), and γH2AX mean fluorescence intensities and area in control and mH2A1 KO cells. The number of cells analyzed for each condition is given (n). Each point corresponds to the mean number of foci per cell, taking from 3 biological replicates, except from γH2AX (2 biological replicates) and HP1α (1 biological replicate). Wilcoxon tests were used to assess the significance of the observed differences. **** $P < 0.0001$, ** $P < 0.01$, * $P < 0.05$, ns: non-significant.
