# Supplementary material for: Recruitment of the Histone Variant MacroH2A1 to the Pericentric Region Occurs upon Chromatin Relaxation and Is Responsible for Major Satellite Transcriptional Regulation
Source: Cells. 2023 Aug 30;12(17):2175. doi: 10.3390/cells12172175 (PMC10486525; doi:10.3390/cells12172175)
Supplement: Supplementary file 1 [file cells-12-02175-s001.zip › Figure S9.pdf]

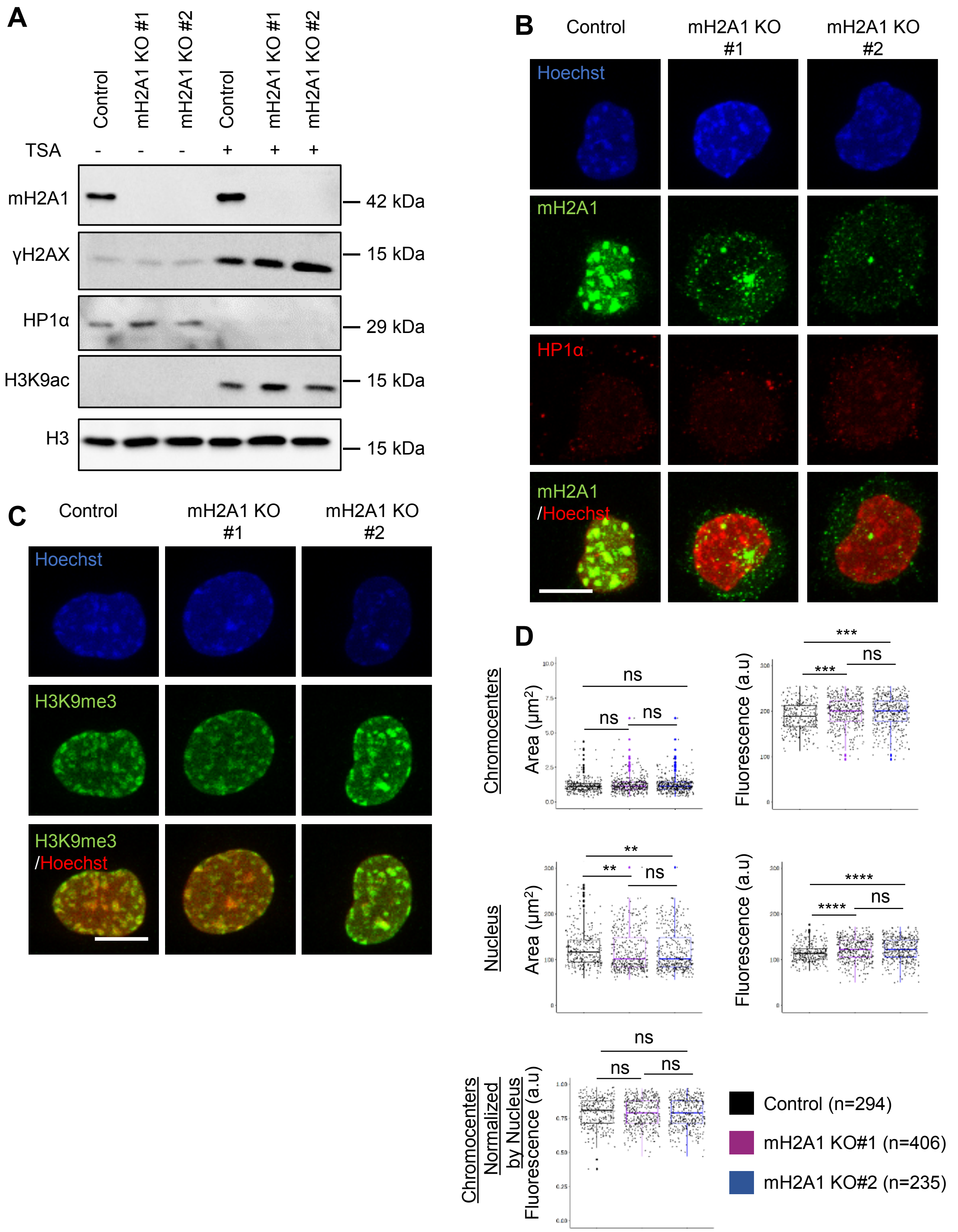

Figure S9. Chromocenter organization in TSA-treated cells in independent of mH2A1. (**A**) Immunoblot analysis for mH2A1, γH2AX, HP1α, H3K9ac and H3 in protein extracts prepared from control and mH2A1 KO cells treated or not with 500 nM of TSA during 48h. Apparent molecular weights are indicated. (**B**) IF confocal images TSA-treated control cells and both mH2A1 KO clones stained with Hoechst and antibodies specific for mH2A1 and HP1α. Scale bar = 10 µm. (**C**) Same as in (B) but cells are stained with Hoechst and antibody specific for H3K9me3. (**D**) Quantification of the mean of chromocenters (Hoechst-dense labelling) and nuclear area and fluorescence intensities in control and mH2A1 KO cells, taken from 3 biological replicates. The last boxplot shows the mean of chromocenter fluorescence normalized by the whole nuclear fluorescence from the same cell. The number of cells analyzed for each condition is given (n). Each point corresponds to the mean number of foci per cell. Wilcoxon tests were used to assess the significance of the observed differences. **** P < 0.0001, ** P < 0.01, * P < 0.05, ns: non-significant.
